# Supplementary material for: Insight into mode-of-action and structural determinants of the compstatin family of clinical complement inhibitors
Source: Nat Commun. 2022 Sep 20;13:5519. doi: 10.1038/s41467-022-33003-7 (PMC9488889; doi:10.1038/s41467-022-33003-7)
Supplement: Supplementary file 1 — Supplementary Information [file 41467_2022_33003_MOESM1_ESM.pdf]

Insight into mode-of-action and structural determinants of the compstatin family of clinical complement inhibitors

Christina Lamers[1,2], Xiaoguang Xue[3], Martin Smieško[1], Henri van Son[3], Bea Wagner[1], Nadja Berger[4], Georgia Sfyroera[4], Piet Gros[3]*, John D. Lambris[4]*, Daniel Ricklin[1]*

[1] Department of Pharmaceutical Sciences, University of Basel, Klingelbergstrasse 50, 4056 Basel, Switzerland; [2] Institute of Drug Discovery, Medical Faculty, Leipzig University, Brüderstr. 34, 04103 Leipzig, Germany; [3] Department of Chemistry, Faculty of Science, Utrecht University, Utrecht, The Netherlands; [4] Department of Pathology & Laboratory Medicine, Perelman School of Medicine, University of Pennsylvania, 401 Stellar Chance, 422 Curie Blvd, Philadelphia, PA 19104, USA

Supplementary Information

Supplementary Materials

Amino acids. The following Fmoc-amino acids were obtained from Chem-Impex (Wood Dale, USA): Fmoc-L-Ala-OH (99.0%), Fmoc-L-Arg(Pbf)-OH (99.1%), Fmoc-L-Asp(OtBu)-OH (99.9%), Fmoc-L-Cys(Trt)-OH (99,1%), Fmoc-L-Gln(Trt)-OH (99.1%), Fmoc-L-Trp(Boc)-OH (99.2%), Fmoc-L-N-Me-Ile-OH (99.9%). Fmoc-L-Glu(OtBu)-OH (98%), Fmoc-L-Gly-OH (98%), Fmoc-L-His(Trt)-OH (98%), Fmoc-L-Ile-OH (98%), Fmoc-Lys(Boc)-OH (98%), Fmoc-L-Ser(tBu)-OH (98%), Fmoc-L-Tyr(tBu)-OH (98%) were obtained from Carbolution (St. Ingbert, Germany). Fmoc-D-Tyr(tBu)-OH (98%), Fmoc-D-Ala-OH and Fmoc-L-(1Me)Trp-OH were obtained from Bachem (Bubendorf, Switzerland), while Fmoc-L-Val-OH was purchased from Sigma-Aldrich (Buchs, Switzerland).

Synthesis of N-alkylated Fmoc-L-tryptophan derivatives. The synthesis of N-alkylated Fmoc-L-tryptophan derivatives was achieved in five steps according to the synthesis scheme shown in Supplementary Fig. 14.

*Synthesis of N-Boc-L-Tryptophan methyl ester (**1**)*
To a mixture of L-Tryptophan methyl ester hydrochloride (500 mg, 1.963 mmol) and NEt_3 (904 µL, 5.889 mmol) in dry DCM (13.5 mL) at 0°C, Boc-anhydride (514 mg, 2.159 mmol) was added in portions. The reaction mixture was stirred at 0°C for 10 min and additional 6 h at rt. After this time the mixture was diluted with DCM and washed with water and brine. The organic layer was dried over Na_2SO_4, filtered and evaporated under reduced pressure. Column chromatography on silica gel (petroleum ether/ethyl acetate 2/1) gave **1** (581 mg, 93%). ^1H NMR (500 MHz, Chloroform-d) δ 1.42 (s, 9H, $OC(CH_3)_3$), 3.28 (q, J = 10.0, 7.5 Hz, 2H, H-1), 3.67 (s, 3H, OCH_3), 4.65 (q, J = 6.1 Hz, 1H, H-2), 5.08 (d, J = 8.2 Hz, 1H, NH, amide), 6.91 – 7.62 (m, 5H, Indol), 8.16 (s, 1H, NH, Indol); ^{13}C NMR (126 MHz, Chloroform-d) δ 27.97 (C-1), 28.31 $OC(CH_3)_3$), 52.21 (OCH_3), 54.16 (C-2), 79.82 ($OC(CH_3)_3$), 110.20, 111.14, 118.72, 119.58, 122.16, 122.69, 127.65, 136.10 (Indol), 155.23 (C=O, Boc), 172.74 (C=O, ester).

*General procedure for synthesis of N-Boc-1-alkyl-L-tryptophan methyl ester derivatives (**2a-c**)*
To a solution of **1** (200 mg, 0.6282 mmol) in DMF (4 ml) at 0°C, NaH (38 mg, 0.8167 mmol, 55% in oil) was added carefully, followed by dropwise addition of alkyl iodide (1.036 mmol). After stirring for 10 min the reaction was quenched with satd. aqueous NH_4Cl solution. The aqueous mixture was extracted with ethyl acetate twice and the combined organic layers were washed twice with water and brine, dried over Na_2SO_4, filtered and evaporated under reduced pressure. The crude product was filtered over a bed of silica gel, rinsed with petroleum ether/ethyl acetate (2/1) and the filtrate was evaporated to dryness to give pure intermediate.

*N-Boc-1-ethyl-L-tryptophan (**2a**)*
2a (161mg, 74%). ^{1}H NMR (500 MHz, Chloroform-*d*) δ 1.37 – 1.49 (m, 12H, CH_3, $OC(CH_3)_3$), 3.27 (t, *J* = 4.8 Hz, 2H, H-1), 3.68 (s, 3H, OCH_3), 4.13 (q, *J* = 7.2 Hz, 2H, CH_2), 4.64 (q, *J* = 6.8, 6.3 Hz, 1H, H-2), 5.05 (d, *J* = 8.2 Hz, 1H, NH), 6.92 - 7.53 (m, 5H, indole).

*N-Boc-1-butyl-L-tryptophan (**2b**)*
1 (200 mg, 0.6282 mmol) in DMF (4 mL) with NaH (38 mg, 0.8167 mmol, 55% in oil) and butyl iodide (118 µL, 1.036 mmol). 1 h, **2b** (254 mg, quant.) ^{1}H NMR (500 MHz, Chloroform-*d*) δ 0.92 (t, *J* = 7.3 Hz, 3H, CH_3), 1.23 – 1.35 (m, 2H, CH_2), 1.43 (s, 9H, $OC(CH_3)_3$), 1.78 (p, *J* = 7.2 Hz, 2H, CH_2), 3.26 (q, *J* = 9.4, 7.4 Hz, 2H, H-1), 3.67 (s, 3H, OCH_3), 4.06 (t, *J* = 7.1 Hz, 2H, CH_2), 4.63 (q, *J* = 6.3 Hz, 1H, H-2), 5.07 (d, *J* = 8.4 Hz, 1H, NH), 6.82 – 7.60 (m, 5H, Indol); ^{13}C NMR (126 MHz, $CDCl_3$) δ 13.63 (CH_3), 20.08 CH_2), 27.90 (C-1), 28.28 ($OC(CH_3)_3$), 32.21 (CH_2), 45.90 (CH_2), 52.10 (OCH_3), 54.22 (C-2), 79.68 ($OC(CH_3)_3$), 109.36, 118.88, 118.92, 121.47, 126.42, 136.12, 155.19, 162.60 (C=O, Boc), 172.72 (C=O, ester).

*N-Boc-1-(3-methylbutyl)-L-tryptophan (**2c**)*
1 (150 mg, 0.4711 mmol) in DMF (3 mL) with NaH (27 mg, 0.6125 mmol, 55% in oil) and 3-methylbutyl iodide (103 µL, 0.7773 mmol). 1 h, **2c** (132 mg 72%). ^{1}H NMR (500 MHz, Chloroform-*d*) δ 0.95 (d, *J* = 6.6 Hz, 6H, CH_3), 1.43 (s, 9H, $OC(CH_3)_3$), 1.56 (dq, *J* = 13.6, 6.6 Hz, 1H, CH), 1.69 (q, *J* = 7.1 Hz, 3H, CH_2), 3.26 (q, *J* = 10.1, 7.7 Hz, 2H, H-1), 3.67 (s, 3H, OCH_3), 4.07 (t, *J* = 7.5 Hz, 2H, CH_2), 4.62 (dd, *J* = 8.8, 5.1 Hz, 1H, H-2), 5.06 (d, *J* = 8.3 Hz, NH), 6.77 – 7.68 (m, 5H, indole); ^{13}C NMR (126 MHz, $CDCl_3$) δ 22.43, 22.46 (CH_3), 25.71 (CH), 27.98 (H-1), 28.36 ($OC(CH_3)_3$), 39.01, 44.45 (CH_2), 52.17 (OCH_3), 54.27 (C-2), 79.75 ($OC(CH_3)_3$), 108.59, 109.38, 119.00, 119.03, 121.56, 126.29, 128.25, 136.14 (indole), 155.25 (C=O, Boc), 172.79 (C=O, ester).

*General procedure for synthesis of 1-alkyl-L-tryptophan methyl ester derivatives (**3a-c**)*
To a mixture of **2a-c** (0.459 mmol) in dry DCM (6 mL), TFA (1.5 mL) was added slowly at 0°C. After stirring for 1.5 h at 0°C, the reaction mixture was diluted with DCM and extracted twice with water. The combined aqueous layers were neutralized with satd. aqueous $NaHCO_3$ solution and extracted twice with ethyl acetate. The combined ethyl acetate layers were dried over Na_2SO_4, filtered and evaporated in vacuo.

*1-ethyl-L-tryptophan (**3a**)*
According to general procedure: **2a** (160 mg, 0.459 mmol) in DCM (6 mL) with TFA (1.5 mL) yield **3a** (78 mg, 70%).

*1-butyl-L-tryptophan (**3b**)*
According to general procedure: **2b** (110 mg, 0.294 mmol) in DCM (4 mL) with TFA (1 mL) gave **3b** (71 mg, 90%).

*1-(3-methyl)butyl-L-tryptophan (**3c**)*
According to general procedure: **2c** (101 mg, 0.260 mmol) in DCM (3 mL) with TFA (650 µL) gave **3c** (20 mg, 27%).

*General procedure for synthesis of Fmoc-1-alkyl-L-tryptophan methyl ester derivatives (**4a-c**)*
To a mixture of **3a-c** (0.317 mmol) in 10% aqueous Na_2CO_3/THF (1:1, 4.8 mL), Fmoc-OSu (117 mg, 0.347 mmol) in THF (1.2 mL) was added dropwise at 0°C. The reaction mixture was stirred at 0°C for 30 min followed by stirring at rt for 20 h. After this time the mixture was diluted with brine and extracted with ethyl acetate (or DCM) twice. The combined organic layers were dried over Na_2SO_4, filtered and concentrated in *vacuo*. Column chromatography on silica gel (PE/EE 4:1) gave intermediate **4a-c**.

*Fmoc-1-ethyl-L-tryptophan (**4a**)*
According to general procedure: **3a** (78 mg, 0.317 mmol) in 10% aqueous Na_2CO_3/THF (1:1, 4.8 mL), Fmoc-OSu (117 mg, 0.347 mmol) in THF (1.2 mL) gave **4a** (107 mg, 72%).

*Fmoc-1-butyl-L-Tryptophan (**4b**)*
According to general procedure: **3b** (69 mg, 0.253 mmol) in 10% aqueous Na_2CO_3/THF (1:1, 4.0 mL), Fmoc-OSu (93 mg, 0.279 mmol) in THF (1.0 mL) gave **4b** (65 mg, 50%). ^{1}H NMR (500 MHz, Chloroform-*d*) δ 0.90 (t, *J* = 7.4 Hz, 3H, CH_3), 1.25 – 1.33 (m, 2H, CH_2), 1.72 – 1.81 (m, 2H, CH_2),

3.23 – 3.37 (m, 2H, H-1), 3.68 (s, 3H, OCH_3), 3.98 – 4.07 (m, 2H, CH_2), 4.19 (t, *J* = 7.2 Hz, 1H, CH-Fmoc), 4.29 – 4.45 (m, 2H, CH_2-Fmoc), 4.73 (dt, *J* = 8.5, 5.4 Hz, 1H, H-2), 5.37 (d, *J* = 8.4 Hz, 1H, NH), 6.83 – 7.88 (m, 14H, indole, C_6H_4); ^{13}C NMR (126 MHz, $CDCl_3$) δ 13.65 (CH_3), 20.09 (CH_2), 27.96 (C-1), 32.23, 45.95 (CH_2), 47.13 (CH-Fmoc), 52.26 (OCH_3), 54.66 (C-2), 66.96 (CH_2-Fmoc), 108.12, 109.50, 118.73, 119.13, 119.91, 120.00, 121.60, 124.68, 125.10, 126.52, 127.00, 127.01, 127.52, 127.63, 136.17, 141.23, 141.46, 143.74, 143.88, 144.33 (indole, C_6H_4), 155.71 (C=O Fmoc), 172.34 (C=O ester).

*Fmoc-1-(3-methyl)butyl-L-tryptophan (**4c**)*
According to general procedure: **3c** (20 mg, 0.069 mmol) in 10% aqueous Na_2CO_3/THF (1:1, 1.0 mL), Fmoc-OSu (26 mg, 0.076 mmol) in THF (500 µL) gave **4c** (28 mg, 80%). ^{1}H NMR (500 MHz, Chloroform-*d*) δ 0.94 (d, *J* = 6.6 Hz, 6H, CH_3), 1.51 – 1.63 (m, 1H, CH), 1.68 (q, *J* = 7.2 Hz, 2H, CH_2), 3.31 (dd, *J* = 5.4, 2.5 Hz, 2H, H-1), 3.68 (s, 3H, OCH_3), 4.02 – 4.12 (m, 2H, CH_2), 4.20 (t, *J* = 7.3 Hz, 1H, CH-Fmoc), 4.28 – 4.48 (m, 2H, CH_2-Fmoc), 4.73 (dt, *J* = 8.3, 5.3 Hz, 1H, H-2), 5.36 (d, *J* = 8.4 Hz, 1H, NH), 6.83 – 7.85 (m, 13H, indole, C_6H_4); ^{13}C NMR (126 MHz, $CDCl_3$) δ 22.38, 22.42 (CH_3), 25.69 (CH), 27.98 (C-1), 38.96, 44.46 (CH_2), 47.14 (CH-Fmoc), 52.27 (OCH_3), 54.68 (C-2), 66.99 (CH_2-Fmoc), 108.23, 109.46, 118.76, 119.15, 119.90, 119.92, 121.63, 125.11, 126.33, 127.01, 127.64, 128.15, 136.13, 141.24, 143.76, 143.89 (20C indole, C_6H_4), 155.72 (C=O Fmoc), 172.34 (C=O ester).

*General procedure for synthesis of Fmoc-1-alkyl-L-tryptophan derivatives (**5a-c**)*
A solution of **4a-c** (0.2476 mmol) and 14% aqueous HCl (270 µL) in dioxane (4 mL) was stirred in a Carius tube at 100°C. After 18 h the reaction mixture was cooled to rt and evaporated under reduced pressure to dryness. Column chromatography on silica gel (DCM/MeOH 98/2) gave pure final products **5a-c**.

*Fmoc-1-ethyl-L-tryptophan (**5a**)*
A solution of **4a** (116 mg, 0.2476 mmol) and 14% aqueous HCl (270 µL) in dioxane (4 mL) was stirred in a Carius tube at 100°C. After 18 h the reaction mixture was cooled to rt and evaporated under reduced pressure to dryness. Column chromatography on silica gel (DCM/MeOH 98/2) gave **5a** 890 mg, 80%). ^{1}H NMR (500 MHz, Methanol-*d*$_4$) δ 1.37 (t, *J* = 7.2 Hz, 3H, CH_3), 3.16 (dd, *J* = 14.7, 8.6 Hz, 1H, H-1a), 3.34 – 3.40 (m, 1H, H-1b), 4.08 – 4.21 (m, 3H, CH_2, CH-Fmoc), 4.24 (dd, *J* = 10.5, 7.0 Hz, 1H, CH_2-Fmoc), 4.31 (dd, *J* = 10.5, 7.3 Hz, 1H, CH_2-Fmoc), 4.51 (dd, *J* = 8.5, 4.9 Hz, 1H, H-2), 7.00 – 7.85 (m, 13H, indole, C_6H_4); ^{13}C NMR (126 MHz, Methanol-*d*$_4$) δ 15.82 (CH_3), 28.69 (C-1), 41.62 (CH_2), 48.36 (CH-Fmoc), 56.45 (C-2), 68.00 (CH_2-Fmoc), 110.30, 110.84, 119.70, 119.85, 120.86, 122.36, 126.26, 126.30, 127.17, 128.13, 128.72, 137.49, 142.52, 145.26 (20 C, indole, C_6H_4), 158.37 (C=O Fmoc), 175.79 (C=O acid).

*Fmoc-1-butyl-L-tryptophan (**5b**)*
According to general procedure: **4b** (60 mg, 0.1208 mmol) and 14% aqueous HCl (131 µL) in dioxane (1 mL), 40 h gave **5b** (14 mg, 24%). ^{1}H NMR (500 MHz, Methanol-*d*$_4$) δ 0.86 (t, *J* = 7.4 Hz, 3H, CH_3), 1.21 – 1.34 (m, 2H, CH_2), 1.74 (q, *J* = 7.2 Hz, 2H, CH_2), 3.15 (dd, *J* = 14.7, 8.7 Hz, 1H, H-1a), 3.37 (dd, *J* = 14.7, 4.8 Hz, 1H, H-1b), 4.05 – 4.18 (m, 3H, CH_2, CH-Fmoc), 4.23 (dd, *J* = 10.5, 7.0 Hz, 1H, CH_2-Fmoc), 4.29 (dd, *J* = 10.5, 7.4 Hz, 1H, CH_2-Fmoc), 4.51 (dd, *J* = 8.7, 4.7 Hz, 1H, H-2), 7.00 – 7.80 (m, 13H, indole, C_6H_4). ^{13}C NMR (126 MHz, MeOD) δ 13.66 (CH_3), 20.12 (CH_2), 27.64 (C-1), 32.23, 46.03 (CH_2), 47.10 (CH-Fmoc), 54.55 (C-2), 67.19 (CH_2-Fmoc), 109.61, 118.72, 119.30, 119.95, 121.73, 125.12, 126.79, 127.05, 127.70, 136.25, 141.27, 143.68, 143.81 (20C, indole, C_6H_4), 155.99 (C=O Fmoc), 175.97 (C=O acid).

*Fmoc-1-(3-methyl)butyl-L-Tryptophan (**5c**)*
According to general procedure: **4c** (66 mg, 0.1293 mmol) and 14% aqueous HCl (140 µL) in dioxane (2 mL), 18 h gave **5c** (48 mg, 75%). ^{1}H NMR (500 MHz, Methanol-*d*$_4$) δ 0.86 (d, *J* = 6.6 Hz, 6H, CH_3), 1.47 (hept, *J* = 6.7 Hz, 1H, CH), 1.60 (q, *J* = 7.3, 6.8 Hz, 2H, CH_2), 3.14 (dd, *J* = 14.7, 8.7 Hz, 1H, H-1a), 3.35 (dd, *J* = 14.7, 4.8 Hz, 1H, H-1b), 4.00 – 4.15 (m, 3H, CH_2, CH-Fmoc), 4.19 (dd, *J* = 10.8, 6.9 Hz, 1H, CH_2-Fmoc), 4.23 – 4.33 (m, 1H, CH_2-Fmoc), 4.47 – 4.56 (m, 1H, H-2), 6.95 – 7.82 (m, 13H, indole, C_6H_4); ^{13}C NMR (126 MHz, MeOD) δ 22.79 (CH_3), 26.77 (CH), 28.62 (C-1), 40.13, 45.21 (CH_2), 48.31 (CH-Fmoc), 56.24 (C-1), 68.02 (CH_2-Fmoc), 110.42, 110.65, 119.67, 119.85, 120.85, 120.89, 122.38, 126.13, 126.23, 126.30, 127.69, 128.10, 128.12, 128.71, 128.73, 129.44, 137.66, 142.48, 145.15, 145.19 (indole, C_6H_4), 158.34 (C=O Fmoc), 175.55 (C=O acid).

Supplementary Tables

Supplementary Table 1. Intermolecular interactions between Cp40 and C3b observed in the crystal structure

Cp40		C3b		Interaction type
**Residue**	**Atom(s)/Group**	**Residue**	**Atom(s)/Group**	
D-Tyr1	Side chain	Val375	Side chain	Lipophilic
	O-H	Ser388	O=C	H-bond
	C=O	Asn390	H-N	H-bond
Ile2	Side chain	Pro347	Side chain	Lipophilic
	Side chain	Ser388	Side chain	Lipophilic
Cys3	N-H	Asn390	O=C	H-bond
Val4	Side chain	Met346	Side chain	Lipophilic
	Side chain	Pro347	Side chain	Lipophilic
	Side chain	Leu454	Side chain	Lipophilic
	Side chain	Arg456	Side chain	Lipophilic
N-MeTrp5	Side chain	Gly345	Cα	Lipophilic
	N-H	Gly345	O=C	H-bond
	Side chain	His392	Side chain	Lipophilic
	methyl	Asn390	Side chain	Lipophilic
	Side chain	Pro393	Side chain	Lipophilic
	C=O	Arg456	H-N	H-bond
Gln6	Side chain N-H	Leu455	O=C	H-bond
	Side chain C=O	Met457	H-N	H-bond
Asp7	none			
Trp8	Side chain	Leu455	Side chain	Lipophilic
	Side chain N-H	Met457	O=C	H-bond
	Side chain	Arg459	Side chain	Lipophilic
	Side chain	Glu462	Side chain	Lipophilic
	Side chain	Asp491	Side chain	Lipophilic
Sar9	Cα	Asp491	Side chain	Lipophilic
Ala10	None			
His11	Backbone N-H	Asp491	Side chain	H-bond
	Side chain N-H	Asp491	Side chain	H-bond
	Side chain	Leu492	Side chain	Lipophilic
Arg12	None			
Cys13	None			
Me-Ile14	None			

Supplementary Table 2. Binding energy of intermolecular lipophilic interaction observed during the MD simulations of compstatin analogs Cp40 and Cp01 bound to β-chain MG core of C3b.

**Cp40**		**Cp01**		Δ (in favor for Cp40)
residue	Binding energy [kcal/mol]	residue	Binding energy [kcal/mol]	[kcal/mol]
sum	**-69.0**	sum	**-62.5**	**-6.5**
D-Tyr1	**-6.0**	Acetyl1	**-0.4**	**-5.6**
Ile2	**-6.2**	Ile2	**-5.6**	**-0.5**
Cys3	**-1.3**	Cys3	**-1.2**	**-0.1**
Val4	**-9.0**	Val4	**-9.1**	**0.1**
1-Me-Trp5	**-15.5**	Trp5	**-14.3**	**-1.2**
Gln6	**-2.8**	Gln6	**-2.8**	**0.1**
Asp7	**-0.9**	Asp7	**-1.2**	**0.3**
Trp8	**-19.0**	Trp8	**-19.8**	**0.7**
Sar9	**-1.1**	Gly9	**-0.9**	**-0.2**
Ala10	**-0.8**	Ala10	**-0.8**	**-0.1**
His11	**-6.2**	His11	**-6.2**	**0.0**
Arg12	**-0.1**	Arg12	**0.0**	**-0.1**
Cys13	**-0.1**	Cys13	**-0.1**	**0.1**
mIle14	**0.0**	Thr14	**0.0**	**0.0**
$CONH_2$15	**0.0**	$CONH_2$15	**0.0**	**0.0**

Supplementary Table 3. Binding energy of intermolecular hydrogen bonds observed during the MD simulations of compstatin analogs Cp40 and Cp01 bound to β-chain MG core of C3b.

**Cp40**		**Cp01**		Δ (in favor for Cp40)
residue	Binding energy [kcal/mol]	residue	Binding energy [kcal/mol]	[kcal/mol]
sum	**-35.7**	sum	**-32.3**	**-3.4**
D-Tyr1	**-6.0**	Acetyl1	**-1.5**	**-4.4**
Ile2	**0.0**	Ile2	**0.0**	**0.0**
Cys3	**-2.4**	Cys3	**-1.9**	**-0.6**
Val4	**0.0**	Val4	**0.0**	**0.0**
1-Me-Trp5	**-4.5**	Trp5	**-5.4**	**0.9**
Gln6	**-4.1**	Gln6	**-3.7**	**-0.4**
Asp7	**-6.3**	Asp7	**-7.3**	**1.0**
Trp8	**-2.9**	Trp8	**-2.4**	**-0.5**
Sar9	**0.0**	Gly9	**0.0**	**0.0**
Ala10	**-2.7**	Ala10	**-3.2**	**0.5**
His11	**-6.5**	His11	**-6.8**	**0.2**
Arg12	**-0.1**	Arg12	**0.0**	**-0.1**
Cys13	**0.0**	Cys13	**0.0**	**0.0**
mIle14	**0.0**	Thr14	**0.0**	**0.0**
$CONH_2$15	**0.0**	$CONH_2$15	**0.0**	**0.0**

Supplementary Table 4. Binding energy of intramolecular hydrogen bonds observed during the MD simulations of compstatin analogs Cp40 and Cp01 bound to β-chain MG core of C3b.

**Cp40**		**Cp01**		Δ (in favor for Cp40)
residue	Binding energy [kcal/mol]	residue	Binding energy [kcal/mol]	[kcal/mol]
sum	**-17.0**	sum	**-22.1**	**5.1**
D-Tyr1	**0.0**	Acetyl1	**-0.3**	**0.3**
Ile2	**0.0**	Ile2	**0.0**	**0.0**
Cys3	**0.0**	Cys3	**-0.3**	**0.3**
Val4	**-4.9**	Val4	**-4.9**	**0.0**
1-Me-Trp5	**-4.9**	Trp5	**-4.9**	**0.0**
Gln6	**-2.1**	Gln6	**-2.1**	**0.0**
Asp7	**-0.9**	Asp7	**-0.8**	**-0.1**
Trp8	**-0.1**	Trp8	**-0.1**	**0.0**
Sar9	**-1.4**	Gly9	**-1.2**	**-0.2**
Ala10	**-0.4**	Ala10	**-0.5**	**0.1**
His11	**-3.5**	His11	**-3.2**	**-0.3**
Arg12	**-2.4**	Arg12	**-2.2**	**-0.2**
Cys13	**-1.3**	Cys13	**-4.4**	**3.1**
mIle14	**0.0**	Thr14	**-2.9**	**2.9**
$CONH_2$15	**0.0**	$CONH_2$15	**-0.1**	**0.1**

Supplementary Table 5. Binding energy of intramolecular lipophilic interactions observed during the MD simulations of compstatin analogs Cp40 and Cp01 bound to β-chain MG core of C3b.

**Cp40**		**Cp01**		Δ (in favor for Cp40)
residue	Binding energy [kcal/mol]	residue	Binding energy [kcal/mol]	[kcal/mol]
sum	**-99.7**	sum	**-84.7**	**-15.0**
D-Tyr1	**-4.5**	Acetyl1	**-0.9**	**-3.6**
Ile2	**-6.0**	Ile2	**-2.2**	**-3.8**
Cys3	**-6.4**	Cys3	**-5.8**	**-0.5**
Val4	**-8.1**	Val4	**-7.8**	**-0.3**
1-Me-Trp5	**-9.3**	Trp5	**-9.5**	**0.2**
Gln6	**-11.9**	Gln6	**-12.1**	**0.2**
Asp7	**-6.1**	Asp7	**-4.6**	**-1.5**
Trp8	**-11.9**	Trp8	**-10.7**	**-1.2**
Sar9	**-4.7**	Gly9	**-2.7**	**-2.0**
Ala10	**-2.9**	Ala10	**-2.8**	**-0.1**
His11	**-6.0**	His11	**-5.8**	**-0.2**
Arg12	**-8.1**	Arg12	**-7.3**	**-0.8**
Cys13	**-8.0**	Cys13	**-6.6**	**-1.4**
mIle14	**-6.0**	Thr14	**-6.0**	**0.0**
$CONH_2$15	**-6.0**	$CONH_2$15	**-6.0**	**0.0**

Supplementary Table 6: Cp40 derivatives synthesized for this study, including sequence, expected and found molecular weight (MW), and purity as determined by analytical HPLC.

Peptide	Sequence[1]	MW calc. [Da]	MW found m/z	Purity[2] [%]
Cp40	yICV(1MeW)QDWSarAHRCmI	1787.8	894.9/2+	93.7
Cp40 y1Y	YICV(1MeW)QDWSarAHRCmI	1787.8	894.9/2+	100.0
Cp40 y1A	AICV(1MeW)QDWSarAHRCmI	1695.8	849.3/2+	97.2
Cp40 y1a	aICV(1MeW)QDWSarAHRCmI	1695.8	849.3/2+	97.9
Cp40 (1MeW)5W	yICVWQDWSarAHRCmI	1773.8	888.2/2+	100.0
Cp40 (1MeW)5(1EtW)	yICV(1EtW)QDWSarAHRCmI	1801.9	902.2/2+	96.9
Cp40 (1MeW)5(1BuW)	yICV(1BuW)QDWSarAHRCmI	1829.9	916.2/2+	97.5
Cp40 (1MeW)5(1MPW)	yICV(1MPW)QDWSarAHRCmI	1843.9	923.2/2+	100.0
Cp40 D7E	yICV(1MeW)QEWSarAHRCmI	1801.9	901.6/2+	99.9
Cp40 D7S	yICV(1MeW)QSWSarAHRCmI	1759.9	881.9/2+	100.0
Cp40 Sar9G	yICV(1MeW)QDWGAHRCmI	1773.8	888.5/2+	79.3[3]
Cp40 R12K	yICV(1MeW)QDWSarAHKCmI	1759.9	881.1/2+	100.0
Cp40 R12Cit	yICV(1MeW)QDWSarAHCitCmI	1788.8	895.4/2+	94.3
Cp40 R12S	yICV(1MeW)QDWSarAHSCmI	1718.8	860.8/2+	96.1
Cp40 mI14T	yICV(1MeW)QDWSarAHRCT	1761.8	881.9/2+	97.4
Cp40 mI14A	yICV(1MeW)QDWSarAHRCA	1731.8	867.1/2+	100.0
Cp40 ΔmI14	yICV(1MeW)QDWSarAHRC	1660.8	831.3/2+	100.0
Cp40 D7S/R12S	yICV(1MeW)QSWSarAHSCmI	1690.8	846.7/2+	94.2

[1]Abbreviations: 1BuW, 1-butyl-L-tryptophan; 1EtW, 1-ethyl-L-tryptophan; 1MeW, 1-Methyl-L-tryptophan; 1MPW, 1-(3-methyl)-propyl-L-tryptophan; Cit, L-citrulline; mI, N-methyl-L-Isoleucine; Sar, L-sarcosine. [2]Purity determined at 214 nm. [3]Derivative coeluted with impurity on reverse phase HPLC.

Supplementary Figures

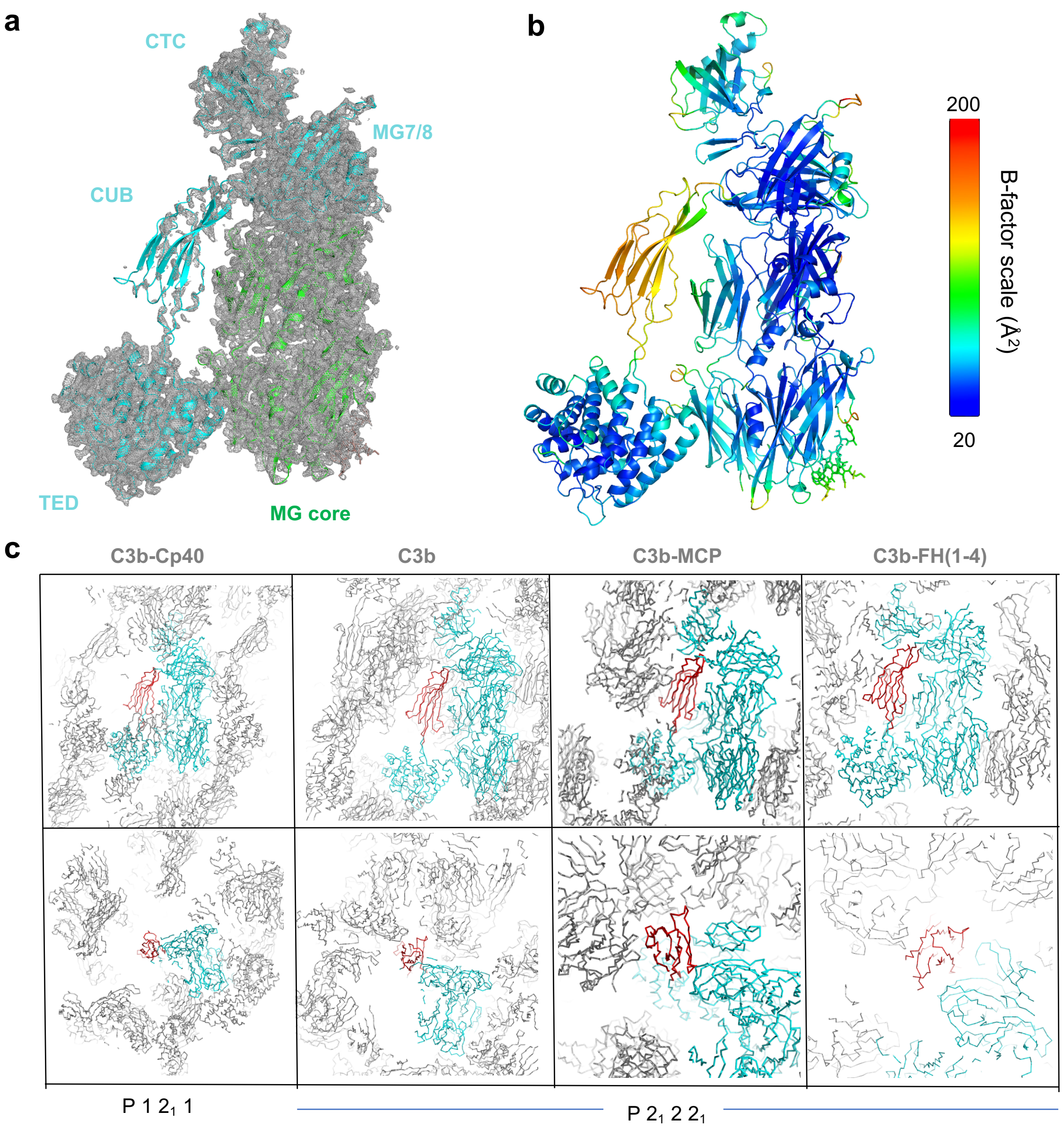

Supplementary Figure 1. Electron density (a) and b-factors (b) of the Cp40-C3b structure. The major domain areas are depicted in panel a with cyan labels marking parts of the α-chain and green labels regions of the β-chain of C3b. c) Comparison of the crystal packing of the C3b-Cp40 structure with the published structures of uncomplexed C3b (PDB 2I07), C3b-MCP (5FO8) and C3b-FH CCP1-4 (2WII). The central C3b is shown in cyan with its CUB domain highlighted in red; crystal mates are shown in grey. CCP, complement control protein domain; MCP, membrane cofactor protein.

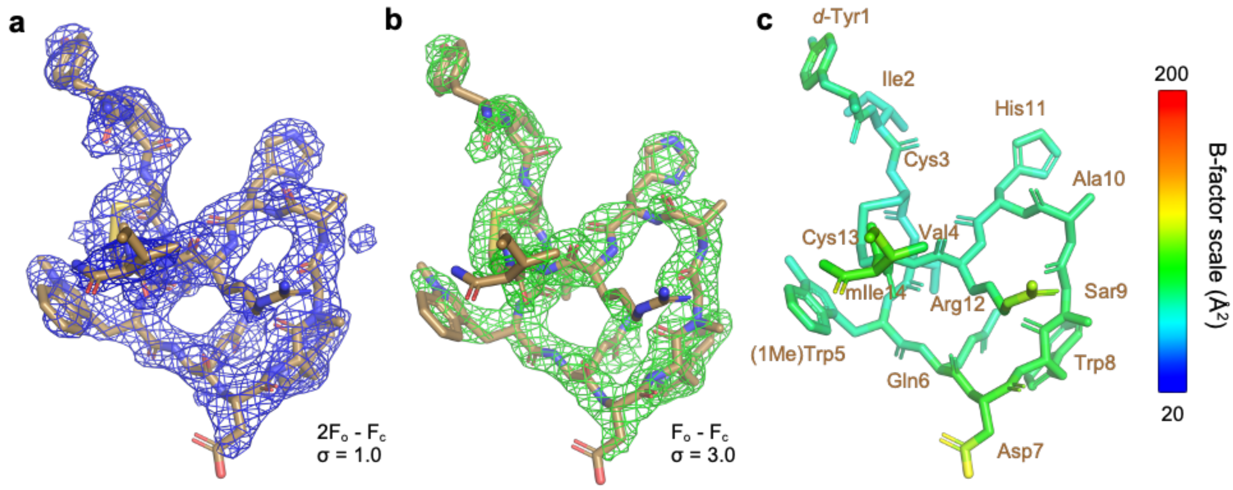

Supplementary Figure 2. Electron density (a), omit map (b) and b-factors (c) of the isolated Cp40 part within the Cp40-C3b structure. The amino acid sequence of Cp40 is indicated in panel c.

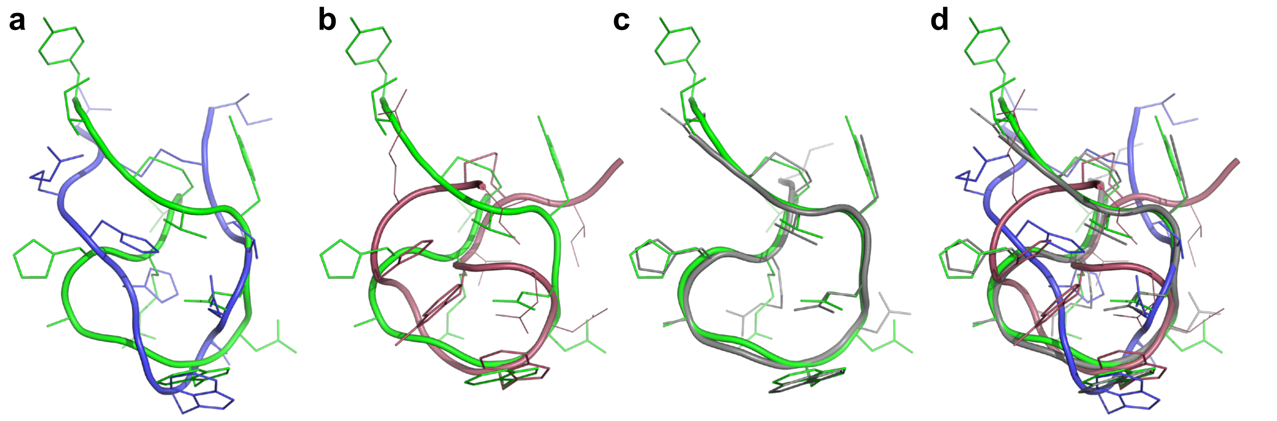

Supplementary Figure 3. Superimposition of the published solution structures of compstatin (a; 1A1P, blue) and Cp10 (b; red) and the bound structure of Cp05 (c; 2QKI, grey) with the C3b-bound structure of Cp40 (green). The main chains are shown as backbone cartoon, the side chains as lines. d) Superimposition of all structures.

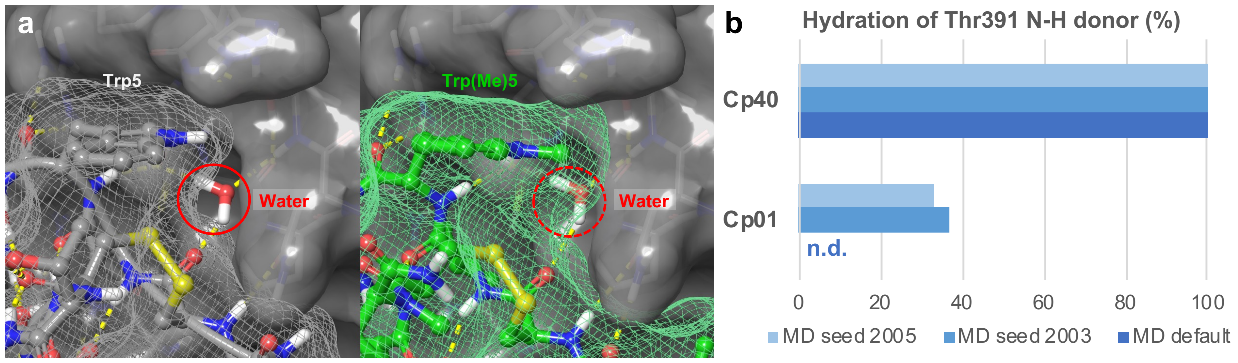

Supplementary Figure 4. Shielding and stabilization of structural water by Trp(Me)5 in Cp40. a) Comparison of the Cp01-C3c (left) and Cp40-C3b (right) structures showing the reduced accessibility of water in the presence of the indole N-methyl group. Solvent-accessible surfaces are depicted as semi-transparent surface for the protein and as mesh for the peptide. b) Residence of water molecules at the Trp(Me)5 site as measured by Thr391 hydration during MD simulations (three seeds). Of note, water molecules were exchanged in Cp01-C3c whereas the same water remained bound in Cp40-C3b throughout the simulation.

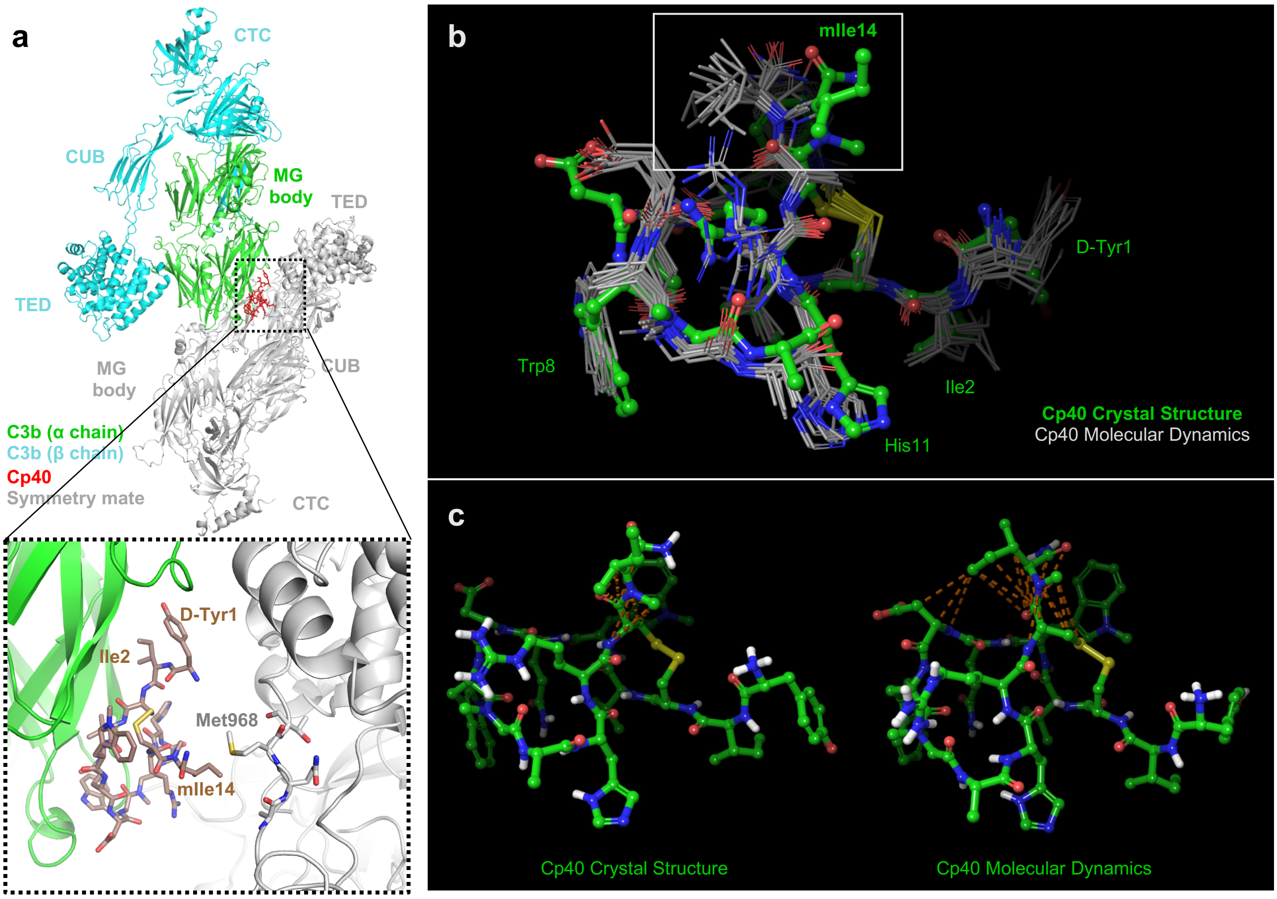

Supplementary Figure 5. Interaction of Cp40 residue mIle-14 with crystal mate in the C3b-Cp40 crystal structure. a) Position of Cp40 between the target C3b and a C3b crystal mate. Close-up view suggesting potential hydrophobic contacts between Ile2 of Cp40 and Met968 of the C3b crystal mate. b) Superimposition of Cp40 extracted from the C3b-Cp40 crystal structure (green) with various poses of the MD simulation of target-bound Cp40 (only peptide part is shown; grey), showing that the extended, solvent-exposed position of mIle14 is unique to the crystal structure. c) During MD simulation, mIle14 engages in notably more intramolecular hydrophobic contacts (brown dashed lines) than suggested by the C3b-Cp40 crystal structure.

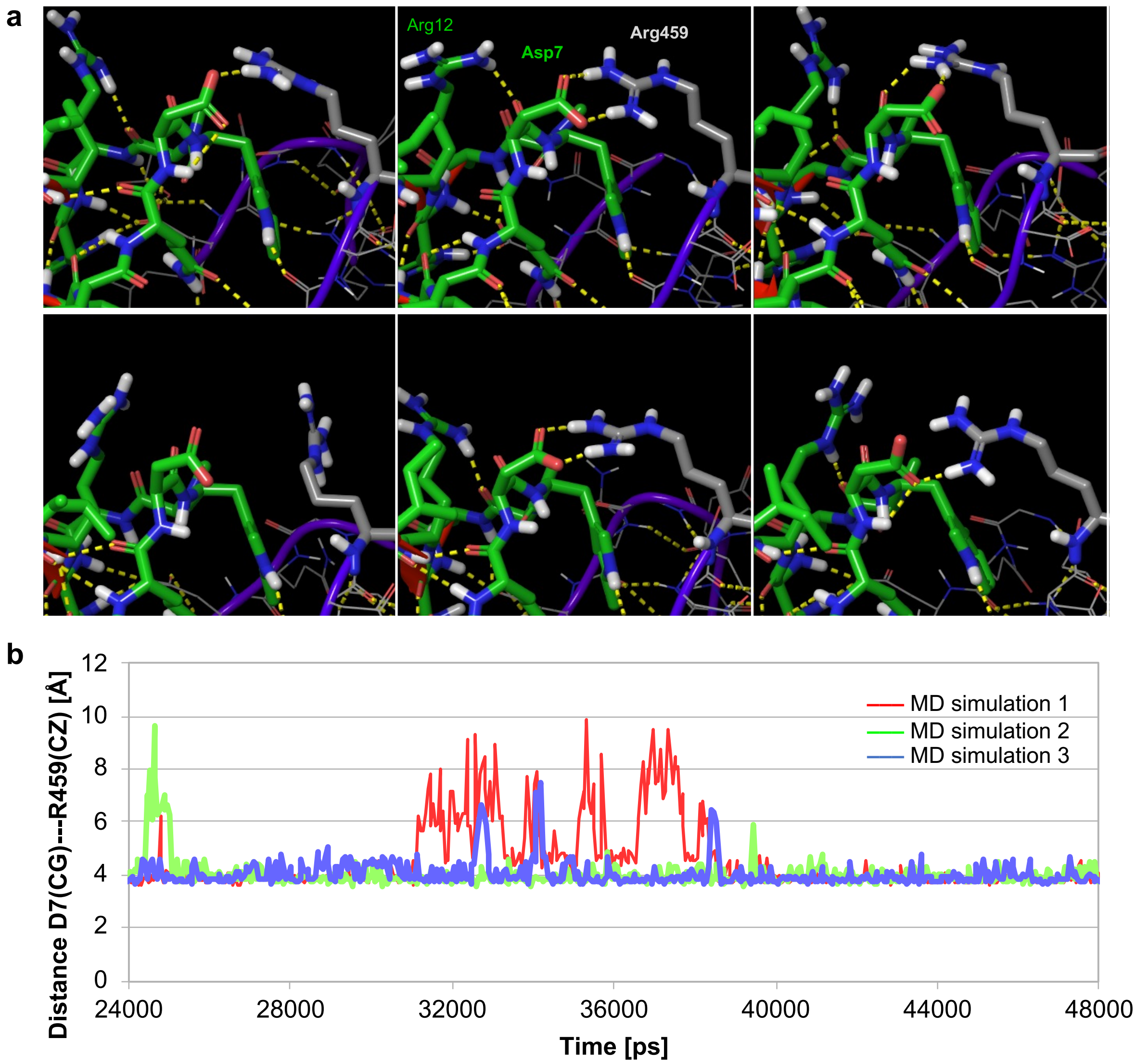

Supplementary Figure 6. Transient electrostatic interaction between Asp7 of Cp40 and Arg459 of C3b as suggested by MD simulations. a) Representative MD frames showing states with the salt bridge fully, partially or not formed. B) Distance measurements between the Cγ atom of Asp4 and the Cζ of Arg459 during three independent MD simulation runs, demonstrating a close proximity between the two residues during a majority of the simulations.

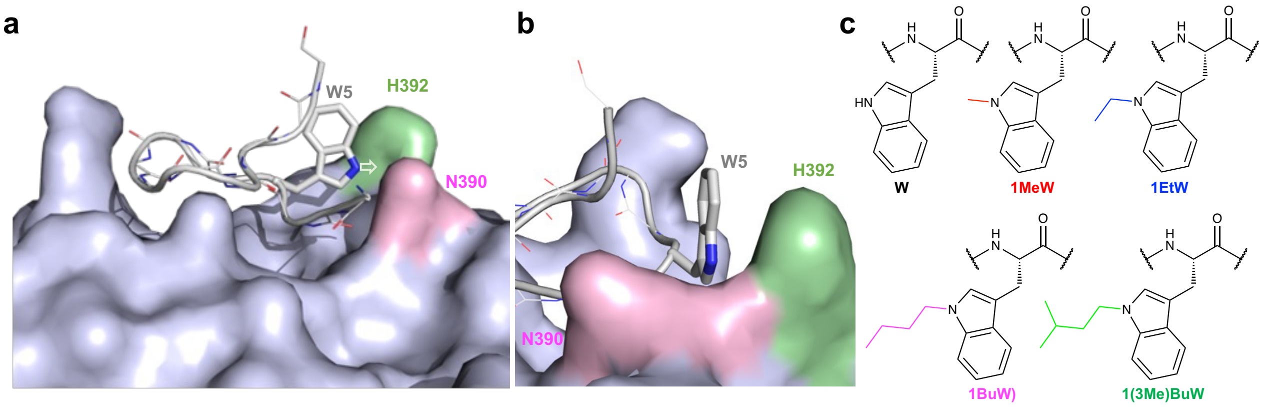

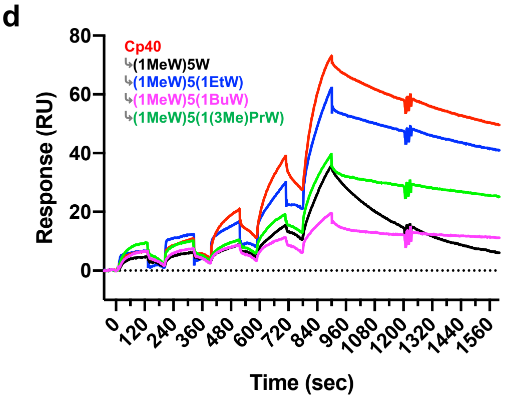

e

Cp40 Analog Position 5	K_D (nM)	k_a (10^6 M^{-1} s^{-1})	k_d (10^{-3} s^{-1})
1MeW (Cp40)	0.8 ± 0.2	1.0 ± 0.4	0.7 ± 0.1
W	21.5 ± 2.1	0.2 ± 0.1	5.1 ± 3.4
1EtW	1.8 ± 0.4	0.7 ± 0.3	1.2 ± 0.3
1BuW	4.1 ± 2.7	0.2 ± 0.0	0.5 ± 0.2
1(3Me)BuW	4.7 ± 0.9	0.2 ± 0.0	0.7 ± 0.0

Supplementary Figure 7. Expansion of the alphatic substituent on the indole nitrogen of (1MeW)5 in Cp01 to address a hydrophobic grove on the C3/C3b/C3c binding site. a-b) Cp01-C3c structure showing the positioning of the W5 indole nitrogen toward a hydrophobic cleft formed by N390, T391 and H392 of C3c. c) Chemical structures of Trp derivatives used at position 5 of Cp40 analogs. d) SPR sensorgrams of Cp40 derivatives containing Trp analogs at position 5. e) Kinetic profiles of Cp40-Trp analogs based on three SPR experiments.

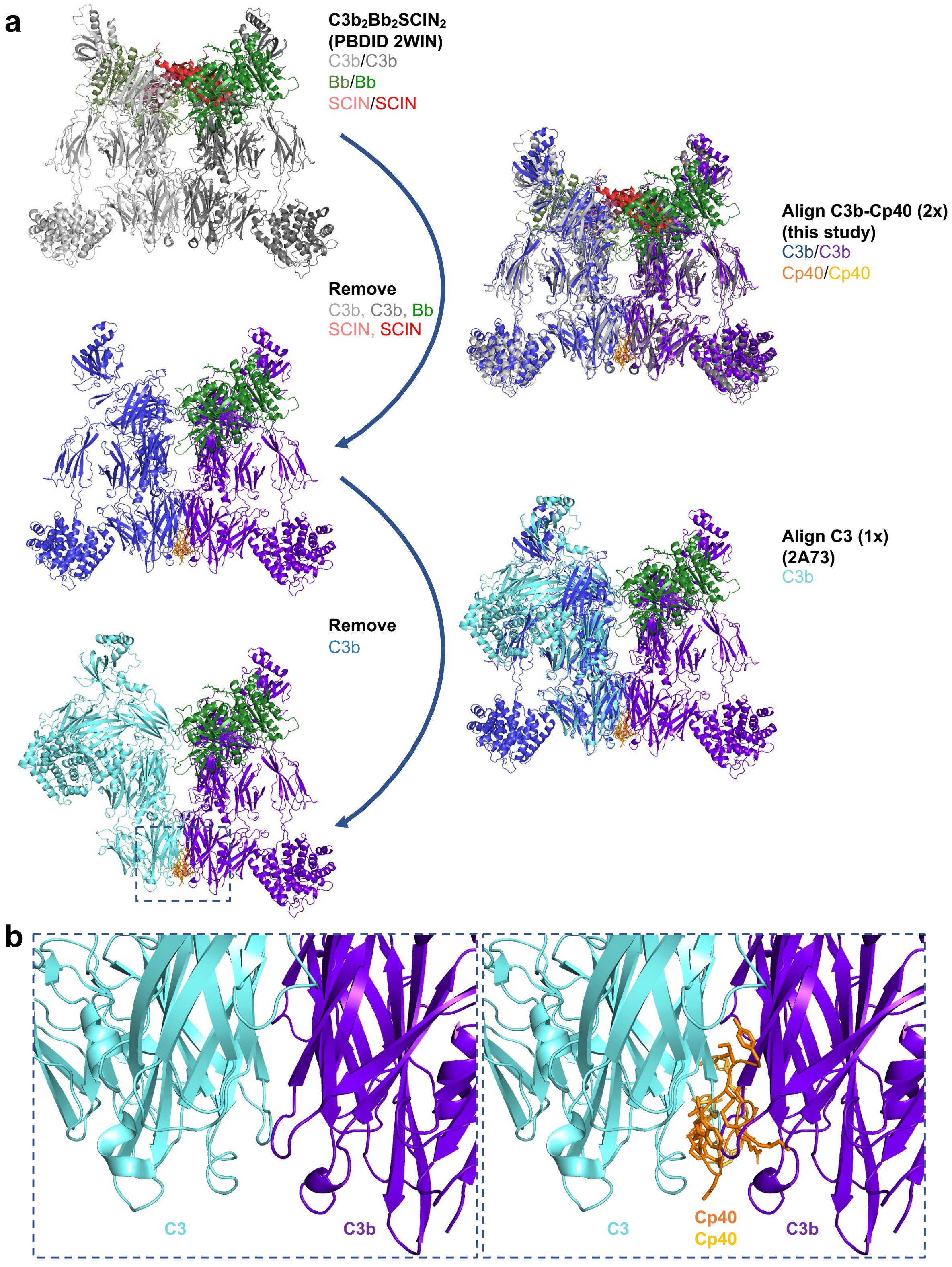

Supplementary Figure 8. Composition of the structural model of the C3:C3bBb interaction, and interference by compstatin analog Cp40, based on the C3b-Cp40 crystal structure of this study and the published structures of C3 (PDBID 2A73) and C3b$_2$Bb$_2$SCIN$_2$ (PDBID 2WIN). a) Assembly of the structural model by stepwise alignment and removal of protein structures using 2WIN as template. b) Closeup view of the proposed C3:C3b dimerization interface (cartoons) in absence (left) and presence (right) of C3-bound and C3b-bound Cp40 (main chains as cartoons, side chains as sticks).

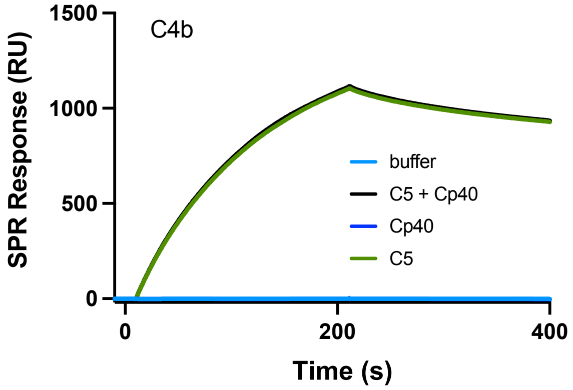

Supplementary Figure 9. Effect of Cp40 on the interaction between soluble C5 (500 nM) and surface-immobilized C4b as assessed by SPR. In the absence of Cp40 binding sites on either C4 or C5, the inhibitor shows no interference with the C5-C4b interaction.

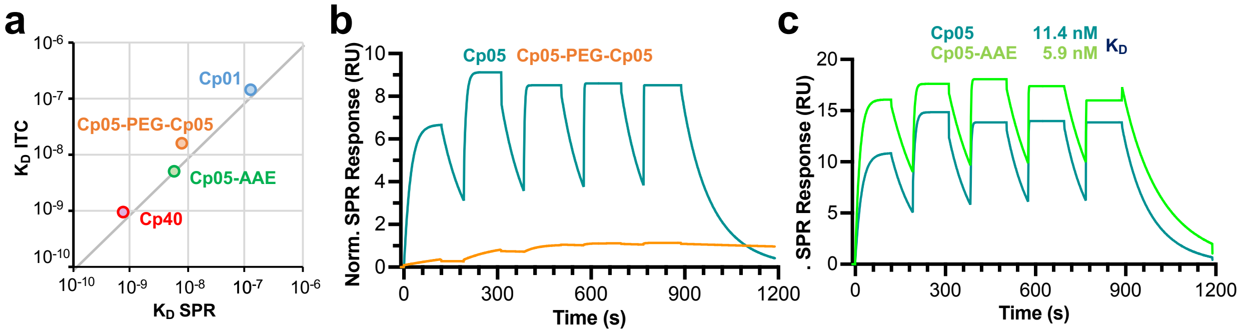

Supplementary Figure 10. Target binding of mono- and bivalent compstatin analogs. a) Correlation between solution (ITC, C3c) and surface (SPR, C3b) binding of compstatin analogs. b) Comparison between monovalent Cp05 and bivalent Cp05-PEG-Cp05 after normalizing the SPR signal by the molecular weight of the analogs, indicating a reduced surface binding capacity of the larger, bivalent derivative. c) Comparison between Cp05 and its Cp05-AAE derivative, which contains an AAE linker to enable attachment to the PEG-40k unit. Whereas the overall binding profiles remained similar, the linker causes an expected increase in SPR signal intensity (molecular weight) and a slight enhancement of binding affinity.

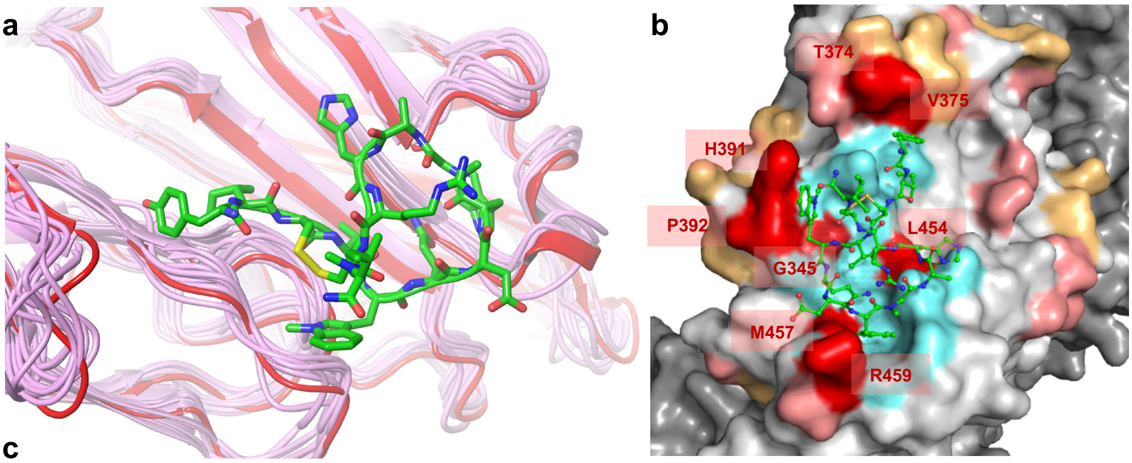

c

```
sp|P01024|CO3_HUMAN                KTPKYFKPGMPFDLMVFVTNPDGSPAYRVPVAVQGEDTVQSLTQGDGVAKLSINTHPSQK  418
tr|A0A2K5VPN1|A0A2K5VPN1_MACFA     KTPKYFKPGMPFDLMVFVTNPDGSPAYRVPVAVQGEDAVQSLTQGDGVAKLSINTHPSQK  418
sp|P01025|CO3_PIG                  KTPKFFKPAMPFDLMVYVTNPDGSPARHIPVVTEDF-KVRSLTQEDGVAKLSINTPDNRN  416
sp|Q2UVX4|CO3_BOVIN                KTPKFFKPAMPFDLMVYVTNPDGSPARHIPVVTQGS-NVQSLTQDDGVAKLSINTQNKRD  416
sp|P12387|CO3_CAVPO                KTPKYFKPAMPFEIMVLVTNPDGSPAPHVPVVTQGS-NVQSLTQADGVARLSINTPNTRQ  416
sp|P01027|CO3_MOUSE                KTPKFFKPAMPFDLMVFVTNPDGSPASKVLVVTQGS-NAKALTQDDGVAKLSINTPNSRQ  418
sp|P01026|CO3_RAT                  KTPKFFKPAMPFDLMVFVTNPDGSPARRVPVVTQGS-DAQALTQDDGVAKLSVNTPNNRQ  417
                                   ****:***.***::** ********* :: *..:.   .::*** ****:**:**  .:.

sp|P01024|CO3_HUMAN                PLSITVRTKKQELSEAEQATRTMQALPYSTVGNSNNYLHLSVLRTELRPGETLNVNFLLR  478
tr|A0A2K5VPN1|A0A2K5VPN1_MACFA     PLSITVRTKKRELSEAEQATRTMEAQPYSTVGNSNNYLHLSVPRAELRPGETLNVNFLLR  478
sp|P01025|CO3_PIG                  SLPITVRTEKDGIPAARQASKTMHVLPYNTQGNSKNYLHLSLPRVELKPGENLNVNFHLR  476
sp|Q2UVX4|CO3_BOVIN                PLTITVRTKKDNIPEGRQATRTMQALPYNTQGNSNNYLHLSVPRVELKPGETLNVNFHLR  476
sp|P12387|CO3_CAVPO                PLSVTVQTKKGGIPDARQAINTMQALPYTTMYNSNNYLHLSMPRTELKPGETINVNFHLR  476
sp|P01027|CO3_MOUSE                PLTITVRTKKDTLPESRQATKTMEAHPYSTMHNSNNYLHLSVSRMELKPGDNLNVNFHLR  478
sp|P01026|CO3_RAT                  PLTITVSTKKEGIPDARQATRTMQAQPYSTMHNSNNYLHLSVSRVELKPGDNLNVNFHLR  477
                                    * :** *:*  :  ..** .**.. **.*  **:******: * **:**:.:**** **

sp|P01024|CO3 HUMAN                MDRAHEAKIRYYTYLIMNKGRLLKAGRQVREPGQDLVVLPLSITTDFIPSFRLVAYYTLI  538
tr|A0A2K5VPN1|A0A2K5VPN1_MACFA     MDRTQEAKIRYYTYLIMNKGKLLKVGRQVREPGQDLVVLPLSITTDFIPSFRLVAYYTLI  538
sp|P01025|CO3_PIG                  TDPGYQDKIRYFTYLIMNKGKLLKVGRQPRESGQVVVVLPLTITTDFIPSFRLVAYYTLI  536
sp|Q2UVX4|CO3_BOVIN                TDPGEQAKIRYYTYMIMNKGKLLKVGRQYREPGQDLVVLPLTITSDFIPSFRLVAYYTLI  536
sp|P12387|CO3_CAVPO                SDPNQEAKIRYYTYLIMNKGKLLKVGRQPREPGQALVVLPMPITKELIPSFRLVAYYTLI  536
sp|P01027|CO3_MOUSE                TDPGHEAKIRYYTYLVMNKGKLLKAGRQVREPGQDLVVLSLPITPEFIPSFRLVAYYTLI  538
sp|P01026|CO3_RAT                  TDAGQEAKIRYYTYLVMNKGKLLKAGRQVREPGQDLVVLSLPITPEFIPSFRLVAYYTLI  537
                                    *  : ****:**::****:***.*** ** ** :*** : ** ::*************
```

Supplementary Figure 11. Species specificity analysis of Cp40. a) Superimposition of the crystal structure of Cp40 (green) with human C3b (dark red) and 10 iterations of a homology model of mouse C3b (light red). b) Visualization of amino acid differences between mouse and human C3b in MG4/MG5 region of C3b. The binding area of Cp40 (green) is shown in cyan. On the surface of human C3b (grey), identical residues are shown in white, homologous residues in orange, and non-homologous in either pale red (outside the Cp40 binding site) or dark red (within the Cp40 binding site). c) Sequence alignment (CLUSTAL Omega) of C3 from human, cynomolgous monkey, pig, cow, guinea pig, mouse and rat, with non-homologous binding site residues highlighted in red.

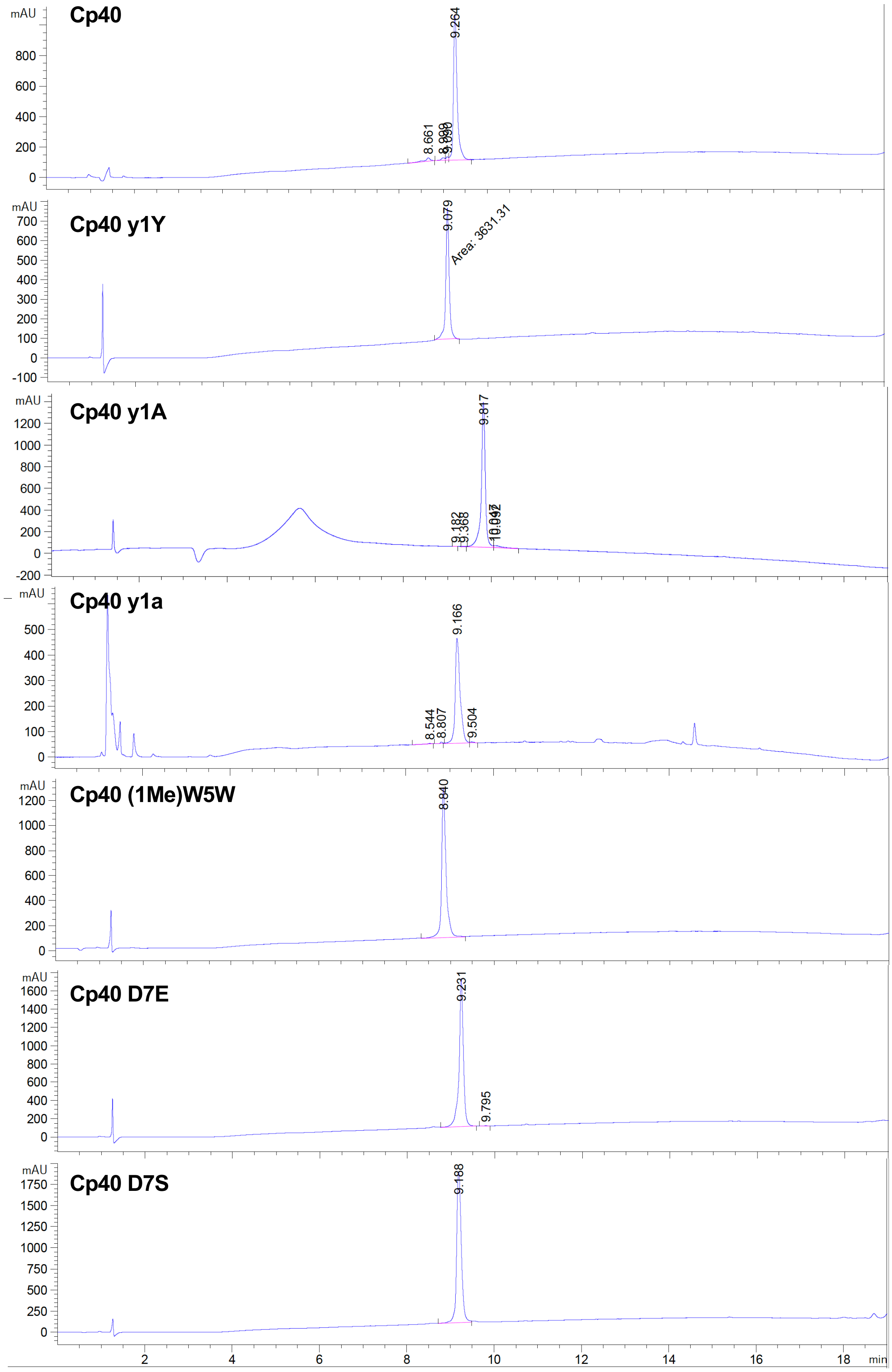

Supplementary Figure 12. HPLC chromatograms of Cp40 derivatives used in this study.

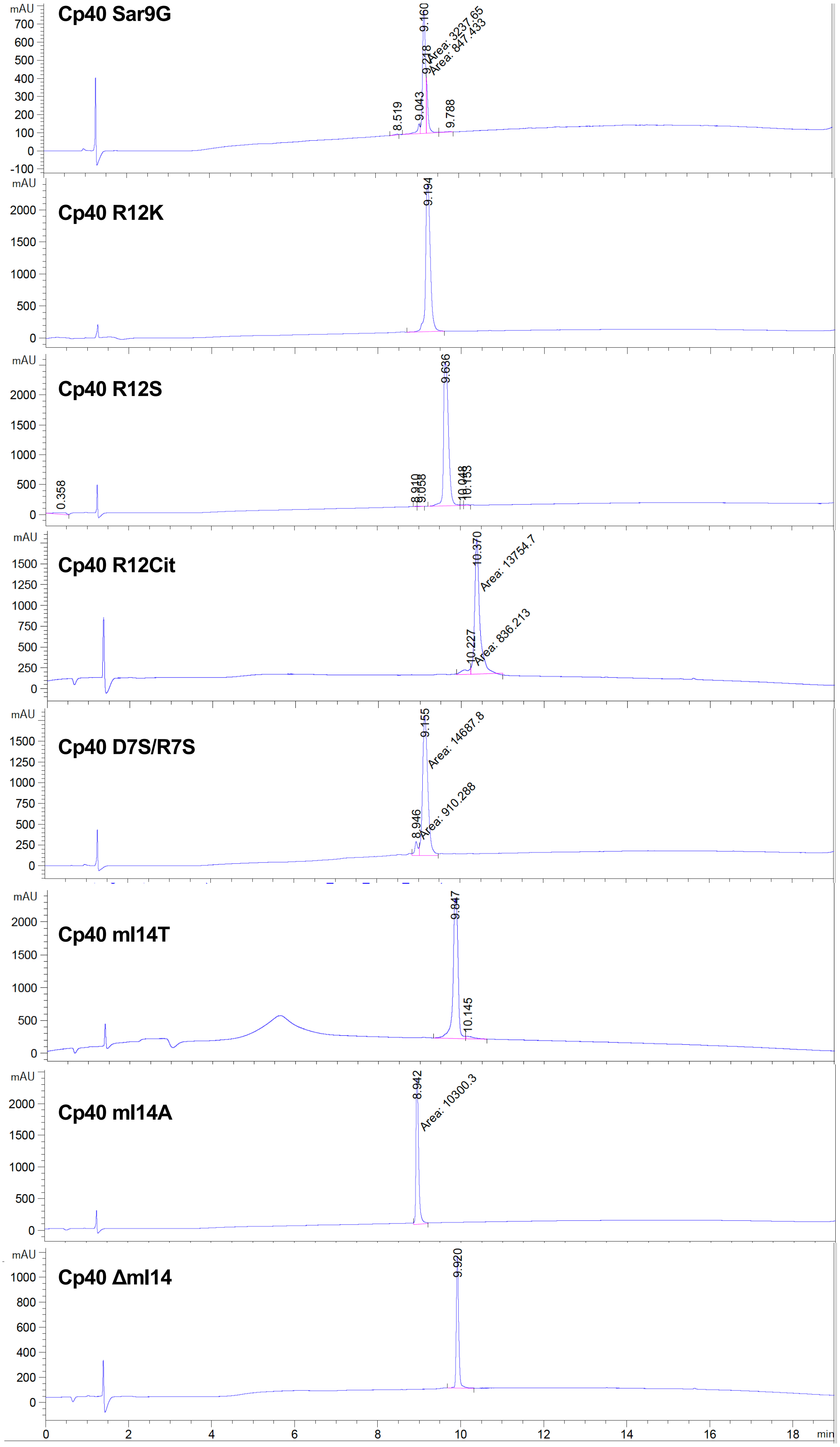

Supplementary Figure 13. HPLC chromatograms of Cp40 derivatives used in this study.

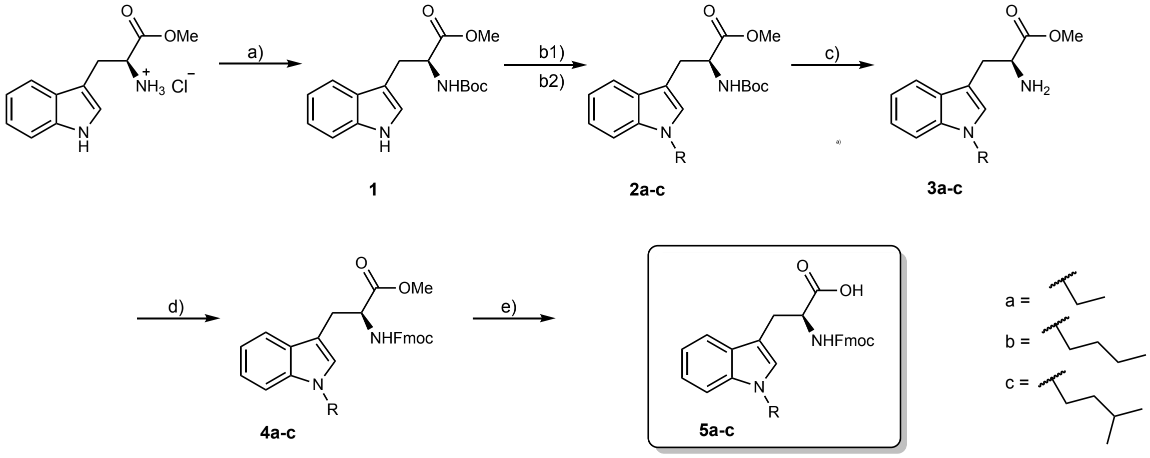

Supplementary Figure 14. Synthesis scheme for the preparation of N-alkylated Fmoc-L-tryptophan derivatives. a) L-Tryptophan methyl ester hydrochloride, $(Boc)_2O$, NEt_3, DCM, 6 h, 0°C – rt [1]; b1) R-I, NaH, DMF, 10 min, 0°C [2]; b2) R-Br, CH_3CN, THF, CsF/celite, overnight, 90°C [4]; c) TFA, DCM, 2 h, 0°C [3]; d) 10% aqueous Na_2CO_3, THF, Fmoc-OSu; overnight, 0°C – rt [5]; e) 14% aqueous HCl, dioxane, overnight, 100°C.
